# A dataset of action potentials recorded from the L5 dorsal rootlet of rat using a multiple electrode array

**DOI:** 10.1016/j.dib.2020.106561

**Published:** 2020-11-21

**Authors:** Benjamin Metcalfe, Alan Hunter, Jonathan Graham-Harper-Cater, John Taylor

**Affiliations:** a Department of Electronic and Electrical Engineering, University of Bath, England; b Department of Mechanical Engineering, University of Bath, England

**Keywords:** Neural interfaces, Bioelectronic medicine, Action potentials, Neural recording, Dermatomes, Dorsal root

## Abstract

This article describes a dataset of action potentials collected from a neural recording experiment conducted on an adult female Sprague Dawley rat. A teased fascicle from the 5^th^ Lumbar dorsal rootlet (L5) was fitted to a custom-made electrode array (10 wire hooks connected as isolated dipoles, with an effective inter-channel spacing of 1 mm) and neural signals were recorded both with and without manual stimulation of the corresponding dermatome. The dataset contains 20 recordings in total, 10 were made with the animal at rest and 10 were made during stimulation. Each recording contains 5 channels of raw voltage data obtained after amplification and digitisation. In [1], a new method was proposed for analysing such multi-channel data in order to automatically identify and classify the action potentials that correspond to dermal afferents. This dataset is of exceptionally high quality for a neural recording and will be useful in both the development and testing of new signal processing methods.

## Specifications Table

.

**Table:** 

Subject	Biomedical Engineering
Specific subject area	Peripheral nerve interfaces for bioelectronic applications.
Type of data	Raw Recordings: MATLAB datafile
How data were acquired	Low noise amplifiers: Digitimer, UK Data Converters: National Instruments DAQ 9222
Data format	Raw
Parameters for data collection	Gain: 80 dB Bandwidth: 10 Hz to 10 kHz Coupling: AC Sample Rate: 5 kHz Electrode Spacing: 1 mm
Description of data collection	The L5 dorsal rootlet was exposed via a laminectomy and a small fascicle was lifted into a recording array formed from wire hooks connected to the amplifiers and data converters. Ten recordings of length 250 ms each were made with the animal at rest and an oscilloscope was used to confirm the presence of neural activity. Ten further recordings also of length 250 ms each were made while the L5 dermatome was stimulated manually. The data files were converted from LabVIEW (TDMS) format to MATLAB (.mat).
Data source location	Institution: University of Bath City/Town/Region: Bath Country: United Kingdom
Data accessibility	Repository name: Mendeley Data Data identification number: 10.17632/ybhwtngzmm.1 Direct URL to data: https://data.mendeley.com/datasets/ybhwtngzmm/1
Related research article	Metcalfe, B., Hunter, A. J., Graham-Harper-Cater, J., & Taylor, J. Array Processing of Neural Signals Recorded from the Peripheral Nervous System for the Classification of Action Potentials. *Journal of Neuroscience Methods*, vo. 347, 108967, Jan. 2021.https://doi.org/10.1016/j.jneumeth.2020.108967

## Value of the Data

• This dataset is important because it provides a high-quality benchmark record of spontaneous neural activity, such recordings are difficult to make and hard to find.
• This dataset will benefit both engineers who are developing new neural interfaces and neuroscientists who wish to build detailed models of the spinal nerve.
• Future developments based on this dataset could include: a new analysis of the action potential morphologies; a benchmark dataset for comparison of action potential classification techniques; teaching materials as an exemplar high quality recording.

## 1 Data Description

**Fig. 1:**
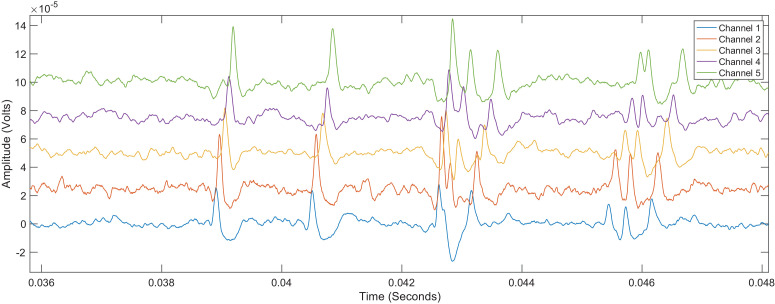
An exemplar data snippet taken from the 1551 recording. Clearly visible are 8 action potentials propagating along the array from Channel 5 (bottom trace) to Channel 1 (top trace). The propagation delays are a function of the underlying conduction velocity and the inter-electrode spacing. The action potentials occurring at approximately 43 ms demonstrate overlapping action potentials with different conduction velocities.

This article describes a neural recording experiment conducted on a single adult female Sprague Dawley rat using a custom-made electrode array and commercial amplifiers and instrumentation [1]. The dataset contains twenty recordings all of length 250 ms with a sampling rate of 50 kHz. The first ten recordings were made with the animal at rest and are located in a folder called “Resting”, the filenames are timestamps that correspond to the time the recordings were started (1551.mat to 1608.mat). The second ten recordings were made while the L5 dermatome was stimulated and are located in a folder called “Cutaneous Stimulation” and likewise the filenames are timestamps that correspond to the time the recordings were started (1554.mat to 1612.mat). The files are all stored as standard MATLAB data files are openable with a wide range of software packages including Python via the SciPy package. Within each data file there is a single matrix called “rawdata” that is a 125,000 × 6 matrix where the columns are as shown in Table 1. Channel 1 represents the most distal electrode, i.e. the closest to the tail. Fig. 1 illustrates a short snippet from the 1551 data file, clearly visible are 8 action potentials propagating from Channel 1 to Channel 5.

**Table 1:** Datafile vector contents.

Column	1	2	3	4	5	6
Contents	Time Vector	Channel 1	Channel 2	Channel 3	Channel 4	Channel 5

## 2 Experimental Design, Materials and Methods

Data were collected from a single adult female Sprague Dawley rat (250 g). The animal was anaesthetised via the intraperitoneal route with 1.5 g/kg of urethane (Sigma) before being placed on a heating mat. A dorsal laminectomy of three of the lumbar spinal vertebrae was performed in order to expose the spinal cord. The individual dorsal roots were then exposed by incision through the dura and the left fifth lumbar dorsal root (L5) was micro-dissected into rootlets/fascicles with fine glass pulled pipettes, in a method described previously [2]. This approach was taken in order to provide access to several small rootlets that contained only a handful of individual axons. A dorsal root was chosen because they are amenable to the micro-dissection technique, and because it is possible to dissect a long enough length to lift into an electrode array. Being purely sensory, the dorsal root should only contain afferent axons that can be activated by direct stimulation of the corresponding dermatome. After dissection, the skin was sutured to an over-hanging rectangular bar, creating a contained pool into which non-conductive mineral oil was poured [1].

The recording array was fabricated on-site and consisted of ten 0.2 mm tungsten wire hooks fed through a polyurethane tube of 0.4 mm internal diameter. The inter-electrode spacing was approximately 0.5 mm, resulting in a total array length of 5 mm. The array was attached to an insulating bar supported by a clamp stand that was positioned over the spinal cord, and a small fascicle (approximately 100 µm in diameter) was gently lifted onto the array. The flexibility of the thin tungsten hooks provided mechanical compliance within the array that ensured good contact between the fascicle and the hooks. The ten electrodes were connected in a shared bipolar configuration whereby each differential amplifier was connected to two adjacent electrodes to give a total of five channels, thus the effective inter-channel electrode spacing was 1 mm. A common reference was used for each amplifier, connected to a 28-gauge hypodermic needle placed subcutaneously over the spinotrapezius muscle.

The amplifiers and filters (Digitimer, UK) were configured for a total gain of 80 dB and a bandwidth of 300–5000 Hz. The amplifier reference was shared throughout the recording system and was also connected to a Faraday cage surrounding the animal. Data acquisition was performed simultaneously on all channels using a bank of analogue-to-digital converters (National Instruments DAQ9222) with a sampling rate of 50 kHz. Throughout the experiment modulation of the neural signals was elicited by stimulating the L5 dermatome via direct cutaneous touch.

## Ethics Statement

All experiments complied with the ARRIVE guidelines and were carried out in accordance with the U.K. Animals (Scientific Procedures) Act, 1986 and associated guidelines.

## CRediT Author Statement

**Benjamin Metcalfe:** Conceptualization, Methodology, Software, Formal analysis, Investigation, Writing – Original Draft. **Alan Hunter:** Methodology, Software, Investigation, Writing – Review & Editing. **Jonathan Graham-Harper-Cater:** Software, Writing – Review & Editing. **John Taylor:** Writing – Review & Editing, Resources.

## Declaration of Competing Interest

The authors declare that they have no known competing financial interests or personal relationships which have, or could be perceived to have, influenced the work reported in this article.

## Data Availability

Action potentials recorded from the L5 dorsal rootlet of rat using a multiple electrode array (Original data) (Mendeley Data).
